# Supplementary material for: Novel antibodies detect nucleocytoplasmic O-fucose in protist pathogens, cellular slime molds, and plants
Source: mSphere. 2025 Feb 6;10(2):e00945-24. doi: 10.1128/msphere.00945-24 (PMC11853108; doi:10.1128/msphere.00945-24)
Supplement: Supplemental material — Figures S1 to S6 and Tables S1 and S2. [file msphere.00945-24-s0001.pdf]

SUPPLEMENT

Novel antibodies detect nucleocytoplasmic O-fucose in protist pathogens, cellular slime molds, and plants

Megna Tiwari, Elisabet Gas-Pascual, Manish Goyal, Marla Popov, Kenjiroo Matsumoto, Marianne Grafe, Ralf Gräf, Robert S. Haltiwanger, Neil Olszewski, Ron Orlando, John Samuelson, and Christopher M. West

Table of Contents

Figure S1. Screening of affinity purified anti-FOT and agnostic antibodies against parasite infected fibroblasts using super-resolution microscopy.

Figure S2. Immunofluorescence analysis of co-labeling of anti-FOS or anti-FOT with anti-PLP6 or AAL in *Toxoplasma* infected fibroblasts.

Figure S3. Immunofluorescence analysis using anti-FOS/T on vegetative stage (growing) *Dictyostelium*.

Figure S4. Mass spectrometric confirmation of 2 fucopeptides from a putative nucleoporin.

Figure S5. Mass spectrometric confirmation of a fucopeptide in a putative helicase/transcription factor.

Figure S6. Mass spectrometric confirmation of fucopeptides from the spore coat protein SP70.

Table S1. Proteins found at increased levels in anti-FOS/T pulldowns of *spy*-KO cells.

Table S2. List of proteomics files available at the ProteomeXchange Consortium via the PRIDE partner repository.

Figure S1. Screening of affinity purified anti-FOT and agnostic antibodies against parasite infected fibroblasts using super-resolution microscopy. (A) HFF host cells containing wild-type RHΔΔ (upper row) or TgSPYΔ (middle row) parasites were probed with rabbit anti-FOT (0.3 µg/ml) and murine anti-SAG1 (1:1000) followed by Alexafluor-488 goat anti-rabbit IgG and Alexafluor-594 goat anti-mouse IgG to localize O-Fuc and outline the parasites, respectively. DAPI (blue) was used to visualize nuclei. Lower row: RHΔΔ-infected fibroblasts were probed with anti-FOT in the presence of 0.2 M alpha-methyl fucose (αMeFuc). Samples were imaged using a Zeiss ELRYA S1 microscope and processed with Super Resolution Structured Illumination Microscopy, and maximum projection images are shown. Scale bars: 5 µm. See Fig. 2 for corresponding probing with anti-FOS. (B, C) Agnostic antibody libraries created from affinity purification of anti-FOT (Thr) or anti-FOS (Ser) (see Fig. 1A) showed no distinguishable labeling in the presence or absence of SPY.

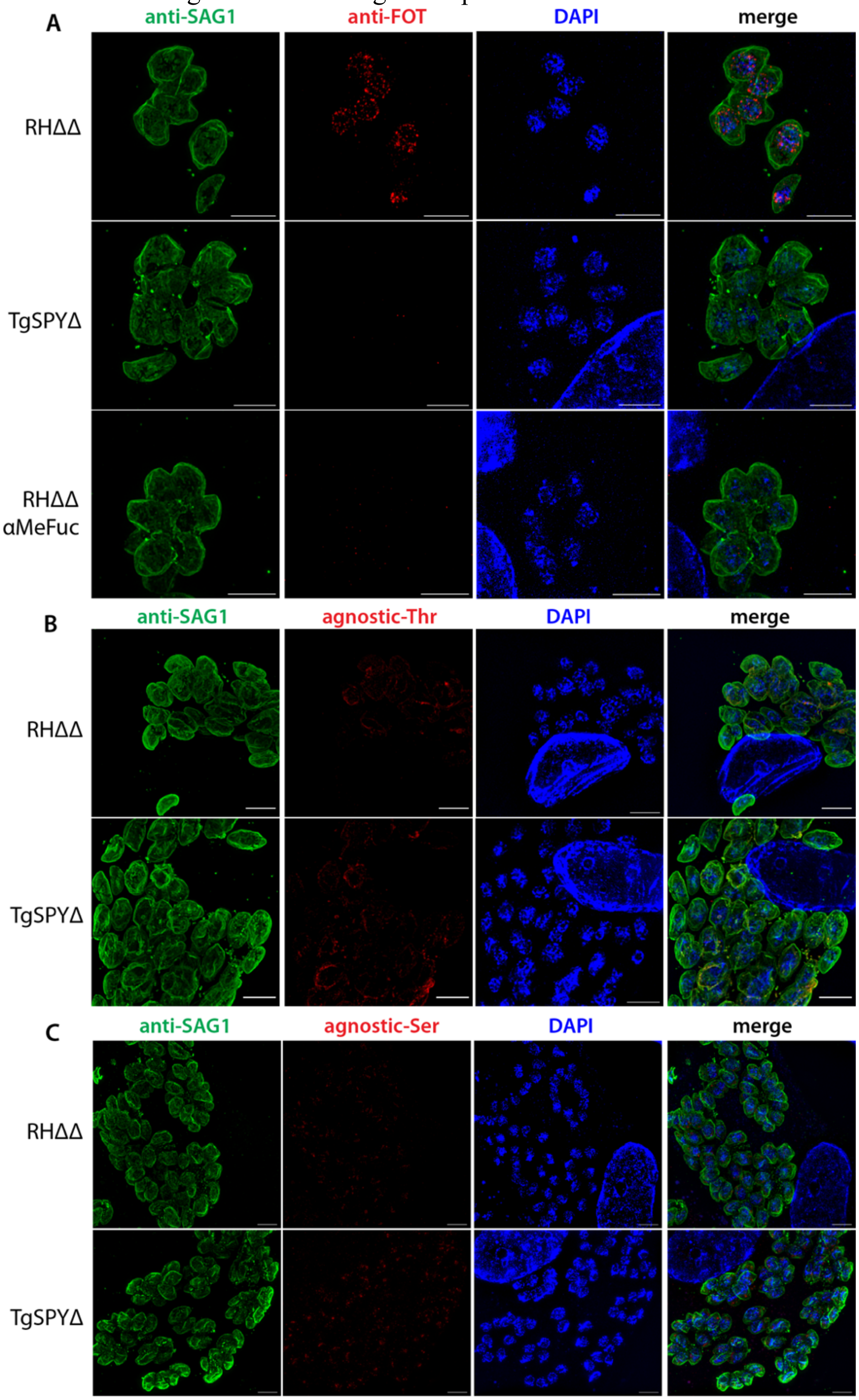

Fig. S2. Immunofluorescence analysis of co-labeling of anti-FOS or anti-FOT with anti-PLP6 or AAL in *Toxoplasma* infected fibroblasts. (A) HFF host cells containing wild-type RHΔΔ were probed with affinity purified rabbit anti-FOS (1 μg/ml) or anti-FOT (0.3 μg/ml) and mouse anti-PLP6 followed by Alexa Fluor-488 goat anti-rabbit IgG and Alexa Fluor-594 goat anti-mouse IgG. (B) Same as above, except that samples were probed with anti-FOS or anti-FOT and AAL. Samples were imaged as in Fig. S1. Scale bars: 5 μm.

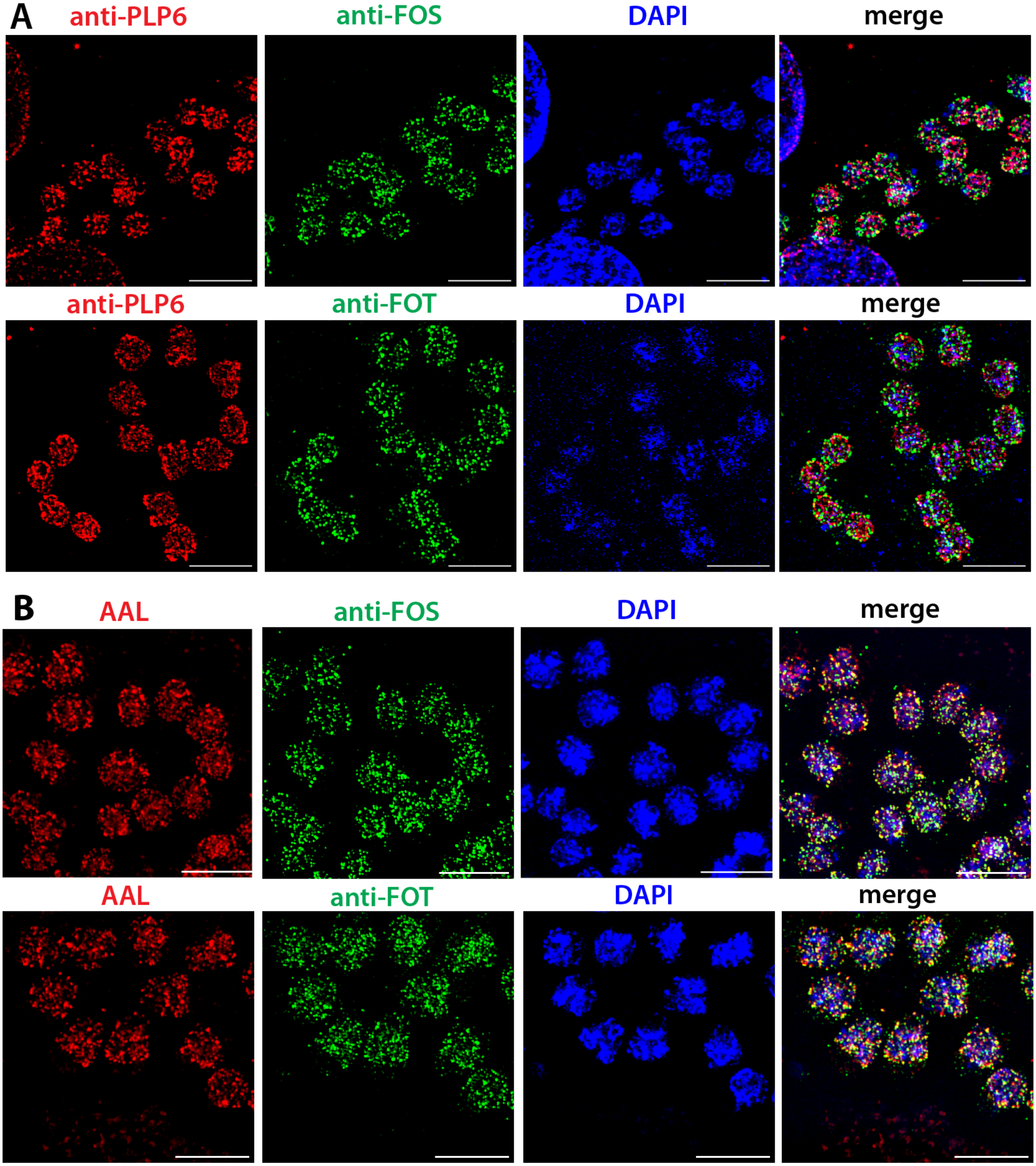

Figure S3. Immunofluorescence analysis using anti-FOS/T on vegetative stage (growing) *Dictyostelium*. Growth stage amoebae were allowed to attach to cover slips and then fixed and permeabilized with MeOH. (A) wild-type (w/t, strain Ax3) amoebae were probed with anti-FOT and AAL-biotin, followed by Alexa Fluor-488 goat anti-rabbit IgG and Alexa Fluor-594 streptavidin. DAPI (blue) was used to visualize nuclei. Maximum projection images are shown. (B) Similarly, w/t, Dd*spy*$^-$ (KO), and Dd*gft*$^-$ amoebae were probed with anti-FOT (0.6 µg/ml) and anti-actin (1:250) followed by Alexa Fluor-488 goat anti-rabbit IgG (1:250) and Alexa Fluor-594 goat anti-mouse IgG (1:250). (C) Same as panel A, except that amoebae were probed with anti-FOS and anti-PLP6 (1:5000), followed by Alexa Fluor-488 goat anti-rabbit IgG and Alexa Fluor-594 goat anti-mouse IgG. (D) Comparison of anti-FOT with nuclear envelope proteins. Amoebae whose Nup62, Nup210, or Src1 genomic loci were C-terminally tagged with mNeon were probed with anti-FOT followed by Alexa Fluor-594 goat anti-rabbit IgG, and co-imaged with intrinsic mNeon fluorescence and DAPI. Single z-slice images are shown. Scale bars: 5 µm.

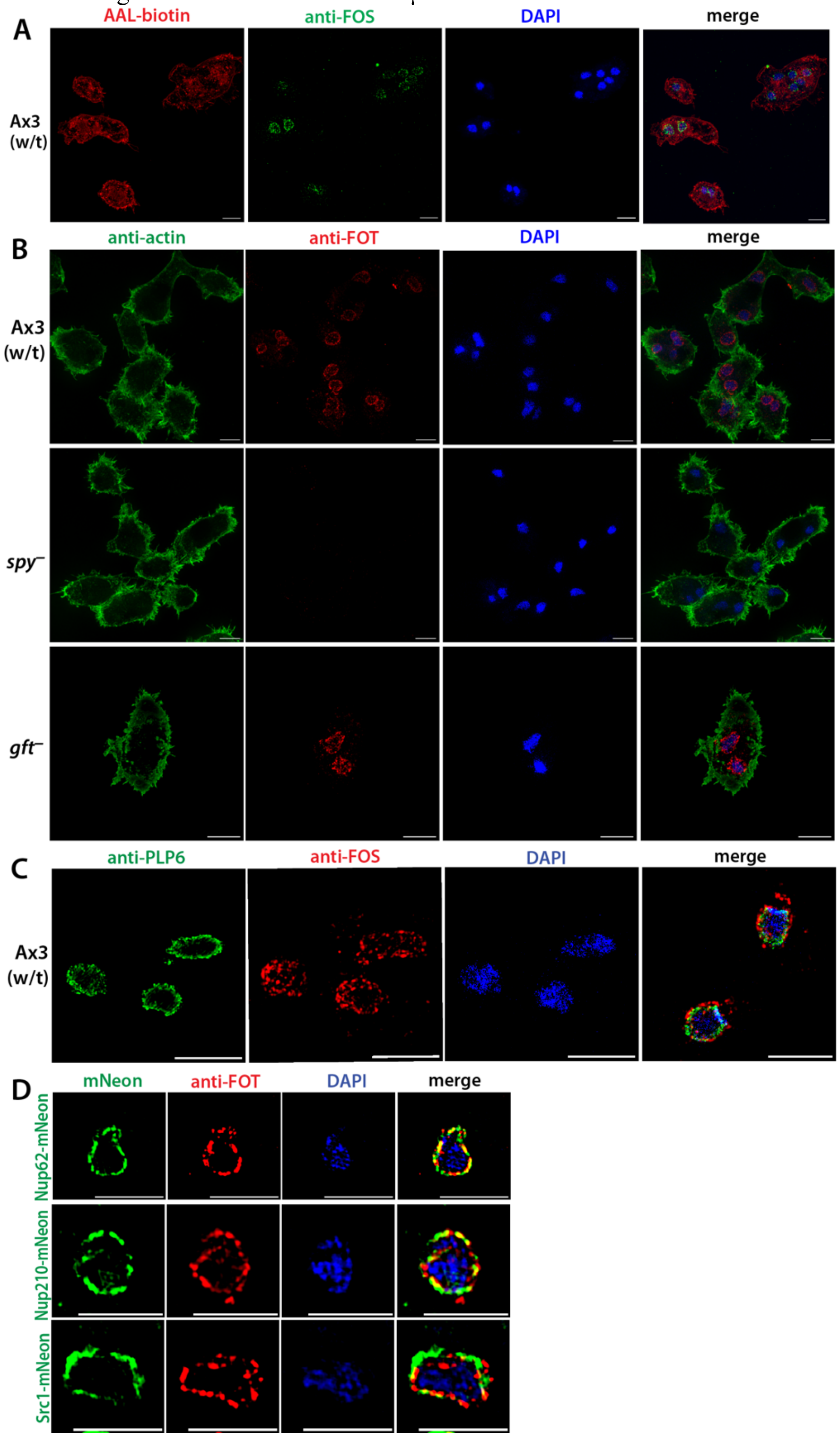

Figure S4. Mass spectrometric confirmation of 2 fucopeptides from a putative nucleoporin (dictyBase DDB_G0274915, Uniprot Q55D2). Peptides from an anti-FOS pulldown from wild-type *Dictyostelium* cells were profiled by nLC on a C18 column and introduced into an Orbitrap mass spectrometer operated in positive ion mode. (A) From vegetative cells, a triply-protonated ion group (a) was detected with a neutral loss of 146, corresponding to a fucose residue. The most abundant isomer (circled in green) was selected for secondary collision (b,c). Calculated and observed *m/z* values for the mono-fucosylated peptide are shown in (a) together with the peptide sequence and attachment site range inferred from analysis of b- and y-ions in panels b and c. Ions detected with loss of 146 are mapped with green arrows.

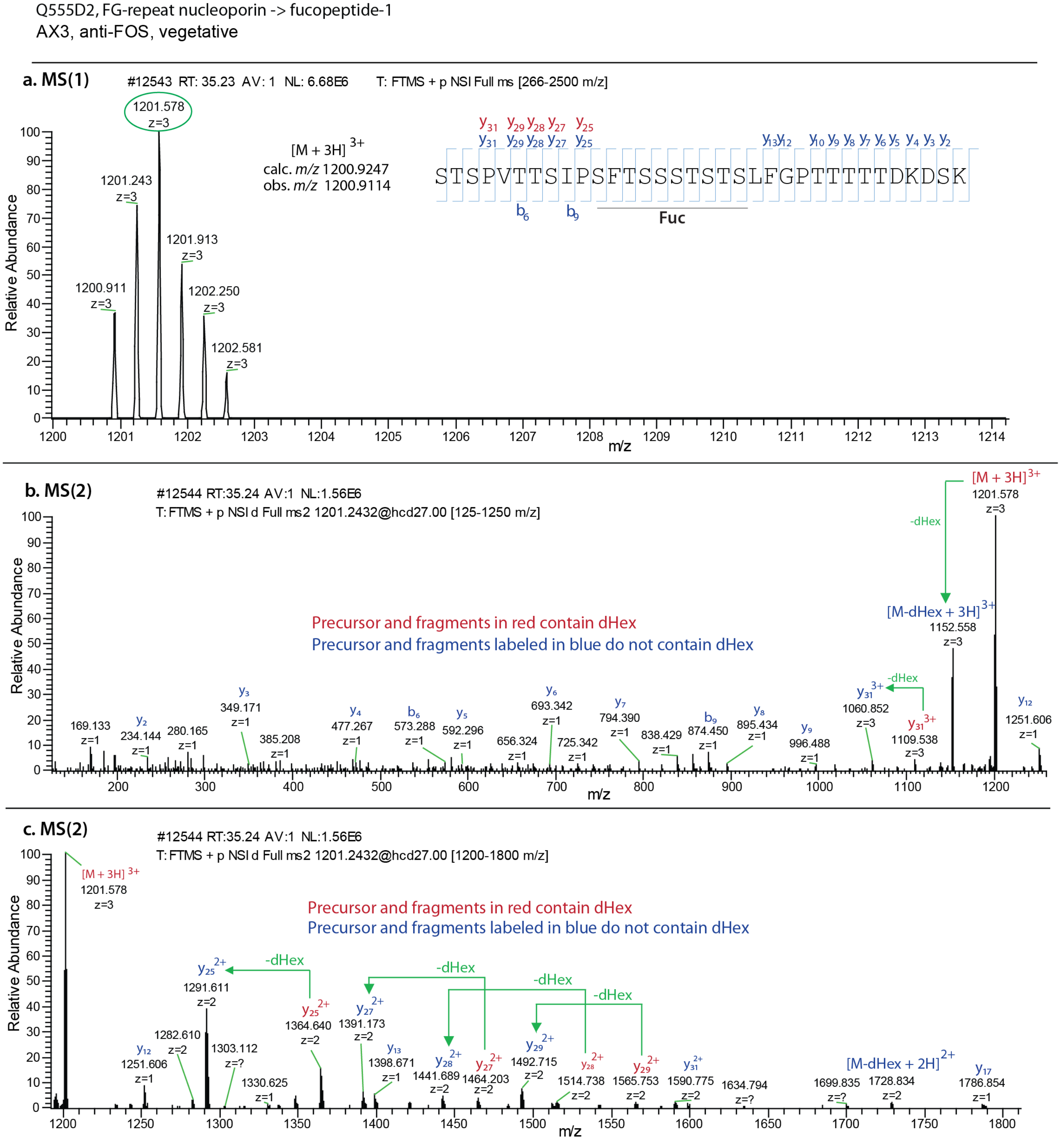

(B) Similarly for a second fucopeptide from the nucleoporin from slug cells. (a) A doubly-protonated ion that was also detected with a neutral loss of 146 was selected for secondary collision. (b) Zoom-in on the parent ion of the MS(2) profile showing the isotopic composition. (c) Zoom-out of the ion profile from which the sequence represented in panel A was deduced. The profile also shows that neutral loss of a fucose residue from the parent ion and y12 and y15 peptides, permitting partial mapping of the fucose to the Ser/Thr rich region of the peptide as indicated.

Q555D2, FG-repeat nucleoporin -> fucopeptide-2
AX3, anti-FOS, slug

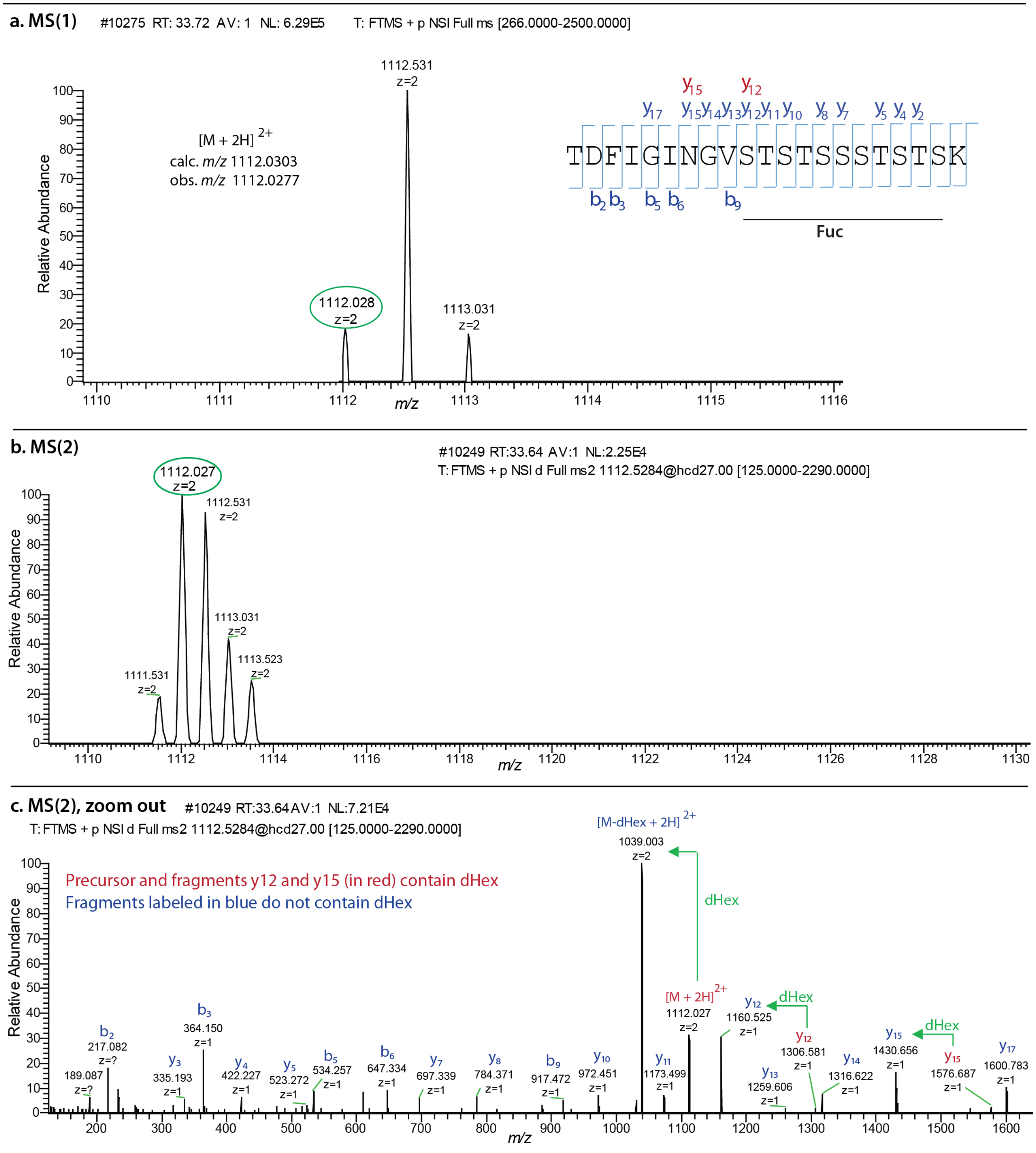

Figure S5. Mass spectrometric confirmation of a fucopeptide in a putative helicase/transcription factor (dictyBase DDB_G0272502, Uniprot Q8T268). Peptides from an anti-FOS pulldown from vegetative wild-type *Dictyostelium* cells were profiled by nLC on a C18 column and introduced into an Orbitrap mass spectrometer operated in positive ion mode. (A) An ion that was also detected with a neutral loss of 146, corresponding to a fucose residue, was selected for secondary collision. (B) MS(2) of the monoisotopic triply-charged parent ion yielded a series of fragment ions allowing assignment of its sequence as presented in panel A. The presence of a deoxyHex on the y25 fragment ion limits its possible location to the region indicated.

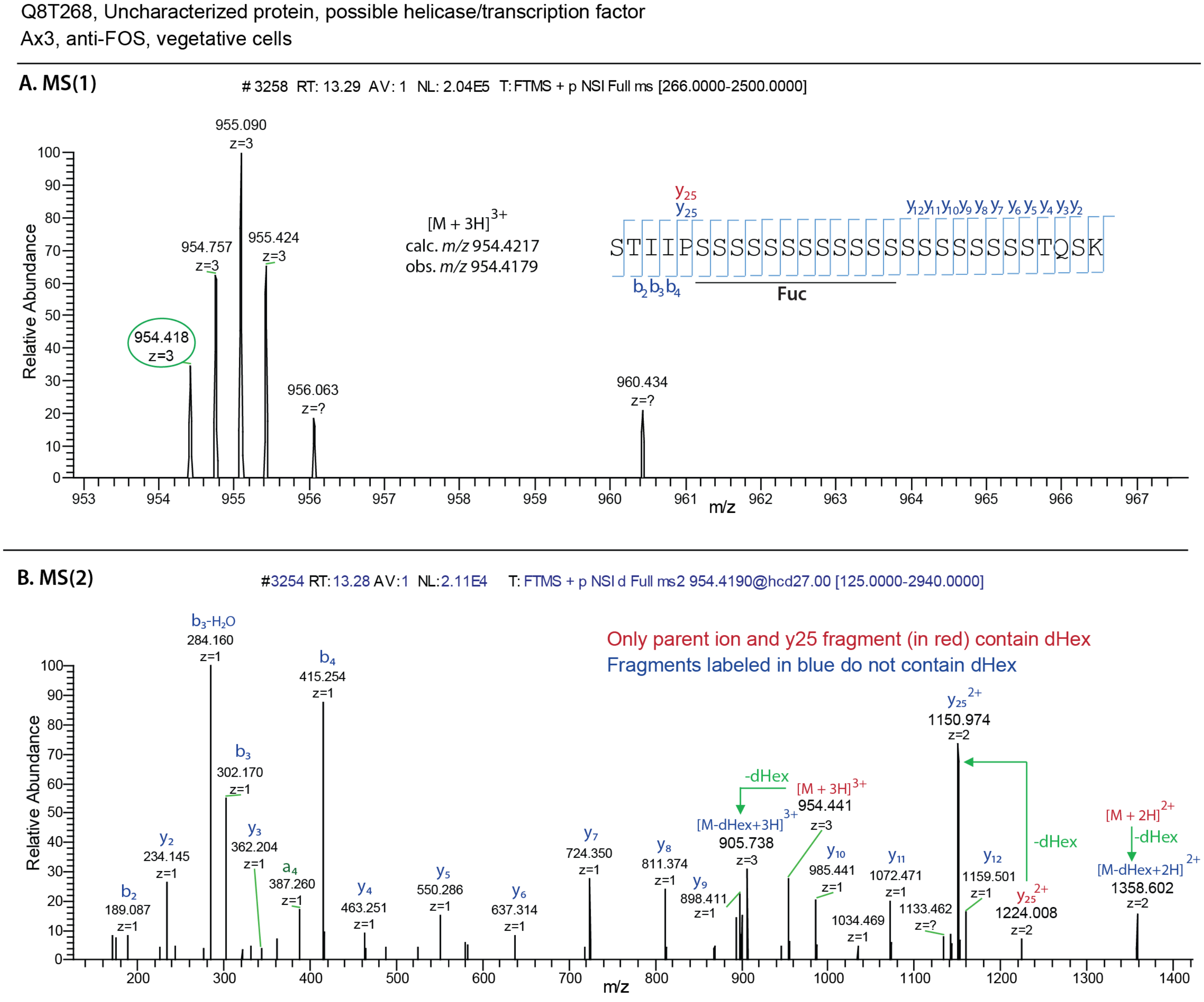

Figure S6. Mass spectrometric confirmation of fucopeptides from the spore coat protein SP70 (dictyBase DDB_G0276761, CotB, Uniprot P15269). Peptides from an anti-FOT pulldown from wild-type *Dictyostelium* slug cells were profiled by nLC on a C18 column and introduced into an Orbitrap mass spectrometer operated in positive ion mode. (A) Summary of findings. (B) An ion that was also detected with a neutral loss of 292, corresponding to 2 fucose residues, was selected for secondary collision. (C) MS(2) of the monoisotopic doubly-charged parent ion yielded a series of fragment ions allowing assignment of its sequence as presented in panel A. The presence of a deoxyHex on the y22 fragment ion limits its possible location to the region indicated. (D) Another ion that was also detected with a neutral loss of 146, corresponding to a single fucose residue, was selected for secondary collision. (E) Same as panel D.

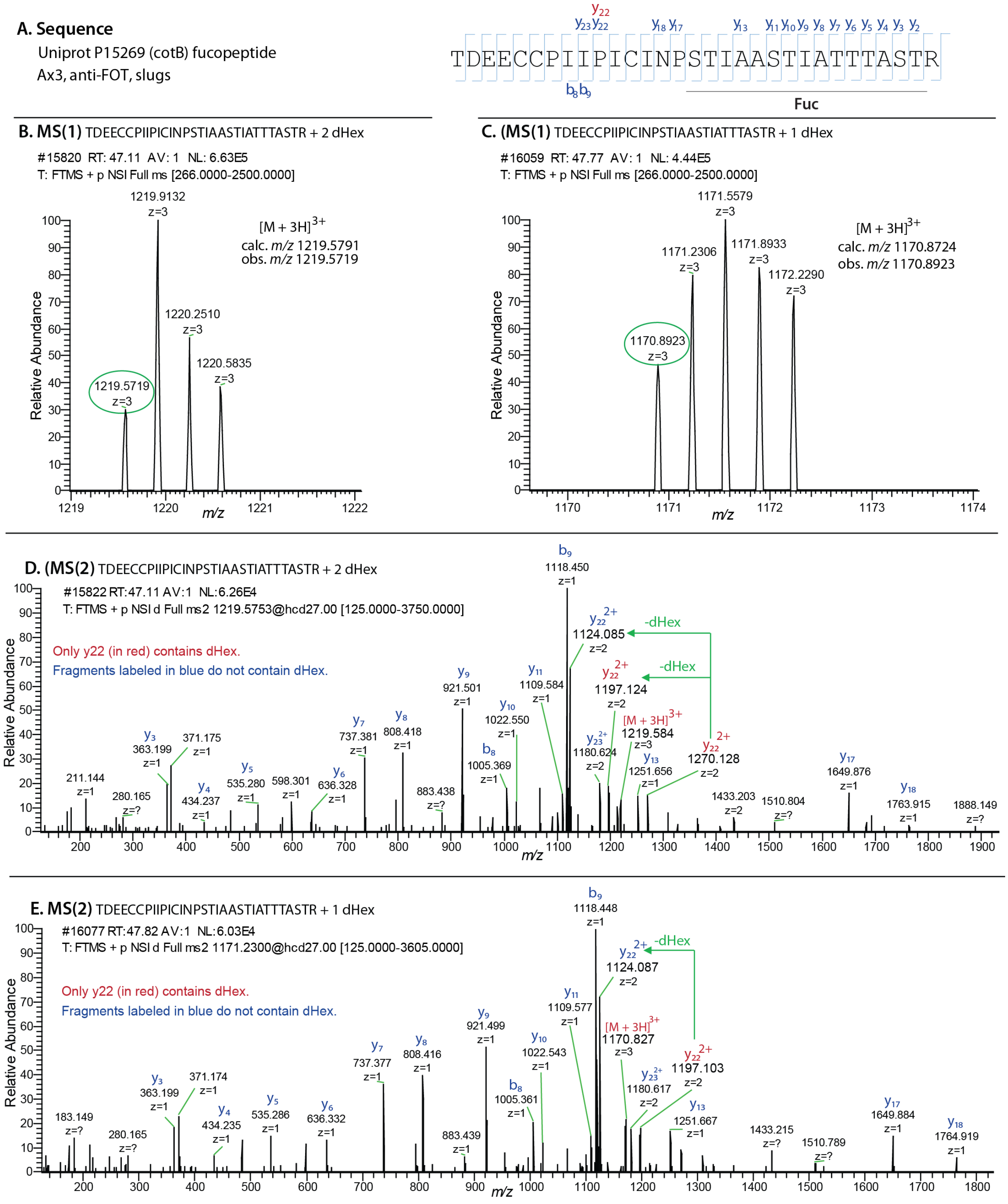

Table S1. Proteins found at increased levels in anti-FOS/T pulldowns of *spy*-KO cells. See volcano plots in Figs. 5E-H and associated text. Format is as in Fig. 6 of the main text. Columns represent fold-change (FC) for abundance based on spectral counting in wild-type vs. *spy*-KO cells using anti-FOS or anti-FOT in vegetative or slug stage cells as indicated. Significances of the fold-changes over the replicates are shown in the adjacent columns. ns, not significantly enriched in both anti-FOS and anti-FOT trials for the indicated stage; nd, not detected.

Accession	DictyBase GeneID	Uniprot Accession	FC FOS Veg	Paired t-test	FC FOT Veg	Paired t-test	FC FOS Slug	Paired t-test	FC FOT Slug	Paired t-test	Short name/description
DDB0216434	DDB_G0290331	Q54G78	0.06	0.003	0.24	0.024	nd		nd		ssrp1, FACT complex subunit SSRP1
DDB0191196	DDB_G0267456	P54653	0.04	0.009	0.12	0.017	ns		ns		cbp2, calcium-binding protein 2
DDB0305759	DDB_G0283653	Q54QT0	0.07	5.14E -07	0.15	0.014	nd		nd		Tyrosinase copper-binding domain protein
DDB0238141	DDB_G0270212	Q58A40	0.15	0.000	0.17	2.85 E-05	ns		ns		Galactose-binding domain-containing protein
DDB0216703	DDB_G0270952	Q55GX8	0.18	3.27E -06	0.10	4.75 E-07	nd		ns		RTE, RetroTransposable Element,Skipper GAG-PRO
DDB0238172	DDB_G0267728	Q55GC4	0.36	0.030	0.08	0.011	nd		nd		uduA3, upreg. in dupA mutant close to UDPA1
DDB0238173	DDB_G0267726	Q55GC5	0.36	0.017	0.13	0.009	nd		nd		uduA1, upregulated in dupA mutant
DDB0191175	DDB_G0285793	P54657	0.23	0.001	0.27	0.002	ns		ns		cadA, calcium-dependent cell adhesion molecule 1
DDB0191525	DDB_G0287587	P54661	0.34	0.005	0.29	0.001	0.09	0.007	0.18	0.013	smlA, small aggregate formation protein
DDB0230179	DDB_G0273017	Q558S7	nd		nd		0.20	0.003	0.14	0.001	isocitrate lyase
DDB0219940	DDB_G0275437	P15649	nd		nd		0.09	0.079	0.08	0.001	7E, HssA/B-like prot. 27-late development

Table S2. List of proteomics files available at the ProteomeXchange Consortium via the PRIDE partner repository.

A. FOS-Veg_All replicates

	.RAW file name
control	SPY_5A_FOS_veg_1_25ul_1Mar24_1.raw
	SPY_5A_FOS_veg_1_25ul_1Mar24_2.raw
	SPY_5A_FOS_veg_1_25ul_1Mar24_3.raw
	SPY_KO_FOS_veg_2_5ul_22Apr24_1.raw
	SPY_KO_FOS_veg_2_5ul_22Apr24_2.raw
	SPY_KO_FOS_veg_2_5ul_22Apr24_3.raw
	SPY_KO_FOS_veg_2_25ul_27Apr24_1.raw
	SPY_KO_FOS_veg_2_25ul_27Apr24_2.raw
	SPY_KO_FOS_veg_2_25ul_27Apr24_3.raw
	SPY_KO_FOS_veg_rep2_1ul_21Jun24_1.raw
	SPY_KO_FOS_veg_rep2_1ul_21Jun24_2.raw
	SPY_KO_FOS_veg_rep2_1ul_21Jun24_3.raw
	SPY_KO_FOS_veg_rep3_0_75ul_24Jun24_1.raw
	SPY_KO_FOS_veg_rep3_0_75ul_24Jun24_2.raw
	SPY_KO_FOS_veg_rep3_0_75ul_24Jun24_3.raw
sample	AX3_4A_FOS_veg_1_5ul_1Mar24_1.raw
	AX3_4A_FOS_veg_1_5ul_1Mar24_2.raw
	AX3_4A_FOS_veg_1_5ul_1Mar24_3.raw
	AX3_FOS_veg_2_75ul_22Apr24_1.raw
	AX3_FOS_veg_2_75ul_22Apr24_2.raw
	AX3_FOS_veg_2_75ul_22Apr24_3.raw
	AX3_FOS_veg_1_5ul_27Apr24_1.raw
	AX3_FOS_veg_1_5ul_27Apr24_2.raw
	AX3_FOS_veg_1_5ul_27Apr24_3.raw
	AX3_FOS_veg_rep2_1ul_24Jun24_1.raw
	AX3_FOS_veg_rep2_1ul_24Jun24_2.raw
	AX3_FOS_veg_rep2_1ul_24Jun24_3.raw
	AX3_FOS_veg_rep3_0_55ul_24Jun24_1.raw
	AX3_FOS_veg_rep3_0_55ul_24Jun24_2.raw
	AX3_FOS_veg_rep3_0_55ul_24Jun24_3.raw

PRIDE	accession	PXD056853
	Results:	AX3_SPY_FOS_veg_4_Bio_5_tech.mzid
	Peaks:	AX3_SPY_FOS_veg_4_Bio_5_tech.mzML

date of submission	15-Oct-24
Submission #	1-20241015-212944-1926934

B. FOT-Veg_All replicates

	.RAW file name
control	SPY_5B_FOT_veg_1_25ul_1Mar24_1.raw
	SPY_5B_FOT_veg_1_25ul_1Mar24_2.raw
	SPY_5B_FOT_veg_1_25ul_1Mar24_3.raw
	SPY_KO_FOT_veg_2ul_23Apr24_1.raw
	SPY_KO_FOT_veg_2ul_23Apr24_2.raw
	SPY_KO_FOT_veg_2ul_23Apr24_3.raw
	SPY_KO_FOT_veg_2_5ul_29Apr24_1.raw
	SPY_KO_FOT_veg_2_5ul_29Apr24_2.raw
	SPY_KO_FOT_veg_2_5ul_29Apr24_3.raw
	SPY_KO_FOT_veg_rep2_0_75ul_21Jun24_1.raw
	SPY_KO_FOT_veg_rep2_0_75ul_21Jun24_2.raw
	SPY_KO_FOT_veg_rep2_0_75ul_21Jun24_3.raw
	SPY_KO_FOT_veg_new_r3_1ul_03Jul24_1.raw
	SPY_KO_FOT_veg_new_r3_1ul_03Jul24_2.raw
	SPY_KO_FOT_veg_new_r3_1ul_03Jul24_3.raw
sample	AX3_4B_FOT_veg_1_5ul_1Mar24_1.raw
	AX3_4B_FOT_veg_1_5ul_1Mar24_2.raw
	AX3_4B_FOT_veg_1_5ul_1Mar24_3.raw
	AX3_FOT_veg_2ul_23Apr24_1.raw
	AX3_FOT_veg_2ul_23Apr24_2.raw
	AX3_FOT_veg_2ul_23Apr24_3.raw
	AX3_FOT_veg_2_5ul_29Apr24_1.raw
	AX3_FOT_veg_2_5ul_29Apr24_2.raw
	AX3_FOT_veg_2_5ul_29Apr24_3.raw
	AX3_FOT_veg_rep2_0_75ul_21Jun24_1.raw
	AX3_FOT_veg_rep2_0_75ul_21Jun24_2.raw
	AX3_FOT_veg_rep2_0_75ul_21Jun24_3.raw
	AX3_FOT_veg_new_r3_1ul_03Jul24_1.raw
	AX3_FOT_veg_new_r3_1ul_03Jul24_2.raw
	AX3_FOT_veg_new_r3_1ul_03Jul24_3.raw

PRIDE	accession	PXD056857
	Results:	
	AX3_SPY_FOT_veg_3_5_Bio_5_tech.mzid	
	Peaks:	
	AX3_SPY_FOT_veg_3_5_Bio_5_tech.mzML	

date of submission	16-Oct-24
Submission #	1-20241016-142622-1926934

C. FOS-Slug_All replicates

	.RAW file name
control	SPY_2A_FOS_slug_4ul_4Mar24_1.raw
	SPY_2A_FOS_slug_4ul_4Mar24_2.raw
	SPY_2A_FOS_slug_4ul_4Mar24_3.raw
	SPY_KO_FOS_slug_3_5ul_18Apr24_1.raw
	SPY_KO_FOS_slug_3_5ul_18Apr24_2.raw
	SPY_KO_FOS_slug_3_5ul_18Apr24_3.raw
	SPY_KO_FOS_slug_4_5ul_25Apr24_1.raw
	SPY_KO_FOS_slug_4_5ul_25Apr24_2.raw
	SPY_KO_FOS_slug_4_5ul_25Apr24_3.raw
	SPY_KO_FOS_slug_new_r1_1_75ul_17Jul24_1.raw
	SPY_KO_FOS_slug_new_r1_1_75ul_17Jul24_2.raw
	SPY_KO_FOS_slug_new_r1_1_75ul_17Jul24_3.raw
sample	AX3_1A_FOS_slug_2ul_29Feb24_0.raw
	AX3_1A_FOS_slug_6ul_5Mar24_1.raw
	AX3_1A_FOS_slug_6ul_5Mar24_2.raw
	AX3_1A_FOS_slug_6ul_5Mar24_3.raw
	AX3_FOS_slug_3_25ul_18Apr24_1.raw
	AX3_FOS_slug_3_25ul_18Apr24_2.raw
	AX3_FOS_slug_3_25ul_18Apr24_3.raw
	AX3_FOS_slug_3_5ul_25Apr24_1.raw
	AX3_FOS_slug_3_5ul_25Apr24_2.raw
	AX3_FOS_slug_3_5ul_25Apr24_3.raw

AX3_FOS_slug_new_r1_1_75ul_18Jul24_1.raw
AX3_FOS_slug_new_r1_1_75ul_18Jul24_2.raw
AX3_FOS_slug_new_r1_1_75ul_18Jul24_3.raw

PRIDE accession PXD056903
Results: AX3_SPY_FOS_slug_4_reps_original.mzid
Peaks:
AX3_SPY_FOS_slug_4_reps_original.mzML

date of submission 16-Oct-24
Submission # 1-20241016-163729-1926934

D. FOT-Slug_All replicates

	.RAW file name
control	SPY_2B_FOT_slug_4_5ul_4Mar24_1.raw
	SPY_2B_FOT_slug_4_5ul_4Mar24_2.raw
	SPY_2B_FOT_slug_4_5ul_4Mar24_3.raw
	SPY_KO_FOT_slug_3_25ul_19Apr24_1.raw
	SPY_KO_FOT_slug_3_25ul_19Apr24_2.raw
	SPY_KO_FOT_slug_3_25ul_19Apr24_3.raw
	SPY_KO_FOT_slug_4_5ul_26Apr24_1.raw
	SPY_KO_FOT_slug_4_5ul_26Apr24_2.raw
	SPY_KO_FOT_slug_4_5ul_26Apr24_3.raw
	SPY_KO_FOT_slug_new_r1_1_5ul_09Jul24_1.raw
	SPY_KO_FOT_slug_new_r1_1_5ul_09Jul24_2.raw
	SPY_KO_FOT_slug_new_r1_1_5ul_09Jul24_3.raw
sample	AX3_1B_FOT_slug_3_5ul_5Mar24_1.raw
	AX3_1B_FOT_slug_3_5ul_5Mar24_2.raw
	AX3_1B_FOT_slug_3_5ul_5Mar24_3.raw
	AX3_FOT_slug_4ul_19Apr24_1.raw
	AX3_FOT_slug_4ul_19Apr24_2.raw
	AX3_FOT_slug_4ul_19Apr24_3.raw
	AX3_FOT_slug_4_75ul_26Apr24_1.raw
	AX3_FOT_slug_4_75ul_26Apr24_2.raw
	AX3_FOT_slug_4_75ul_26Apr24_3.raw
	AX3_FOT_slug_new_r1_1_25ul_09Jul24_1.raw
	AX3_FOT_slug_new_r1_1_25ul_09Jul24_2.raw
	AX3_FOT_slug_new_r1_1_25ul_09Jul24_3.raw

PRIDE accession PXD056866
Results:
AX3_SPY_FOT_slug_4_original_prep_runs.mzid
Peaks:
AX3_SPY_FOT_slug_4_original_prep_runs.mzML

date of submission 16-Oct-24
Submission # 1-20241016-182054-1926934

E. FOS/T-Veg_Spy overexpression replicates

	.RAW file name	
SPY_KO (3)	SPY_5A_FOS_veg_1_25ul_1Mar24_1.raw	FOS
	SPY_5A_FOS_veg_1_25ul_1Mar24_2.raw	
	SPY_5A_FOS_veg_1_25ul_1Mar24_3.raw	
	SPY_5B_FOT_veg_1_25ul_1Mar24_1.raw	FOT
	SPY_5B_FOT_veg_1_25ul_1Mar24_1.raw	
	SPY_5B_FOT_veg_1_25ul_1Mar24_1.raw	
SPY_OE (2)	sample_6A_FOS_T_3_5ul_8Mar24_1.raw	FOS
	sample_6A_FOS_T_3_5ul_8Mar24_2.raw	
	sample_6A_FOS_T_3_5ul_8Mar24_3.raw	
	sample_6B_FOS_T_3_5ul_8Mar24_1.raw	FOT
	sample_6B_FOS_T_3_5ul_8Mar24_2.raw	
	sample_6B_FOS_T_3_5ul_8Mar24_3.raw	
AX3 (1)	AX3_4A_FOS_veg_1_5ul_1Mar24_1.raw	FOS
	AX3_4A_FOS_veg_1_5ul_1Mar24_2.raw	
	AX3_4A_FOS_veg_1_5ul_1Mar24_3.raw	
	AX3_4B_FOT_veg_1_5ul_1Mar24_1.raw	FOT
	AX3_4B_FOT_veg_1_5ul_1Mar24_1.raw	
	AX3_4B_FOT_veg_1_5ul_1Mar24_1.raw	

PRIDE accession PXD057018
Results: AX3_SPY_OE_KO_veg_cells_FOS_T_rep1.mzid
Peaks: AX3_SPY_OE_KO_veg_cells_FOS_T_rep1.mzML

date of submission 21-Oct-24
Submission # 1-20241021-153215-1926934
